# Sequence motifs capable of forming DNA stem–loop structures act as a replication diode

**DOI:** 10.1002/2211-5463.12233

**Published:** 2017-06-04

**Authors:** Andrey Shirak, Uri Seroussi, Elisha Gootwine, Eyal Seroussi

**Affiliations:** ^1^ Institute of Animal ScienceAgricultural Research Organization (ARO)Rishon LeTsiyonIsrael; ^2^ Department of NeurobiologyWise Faculty of Life Sciences and Sagol SchoolTel Aviv UniversityIsrael

**Keywords:** DNA loop structure, DNA stability, DNA thermodynamics, microRNA, orientation‐dependent block

## Abstract

Calculating peak‐height ratios between single‐nucleotide polymorphisms (SNP) alleles in sequencing chromatograms is a practical method for estimating their copy number proportions (CNPs). However, it is surprising that sequencing DNA from different directions might yield different results. We analyzed three adjacent SNPs within the ovine period circadian‐clock 2 (*PER2*) gene that displayed such behavior. We compared Sanger and DNA‐seq sequencing for this locus and applied high‐resolution melt and MFOLD analyses to point to the DNA secondary structure that underlined this phenomenon. A synthetic system of oligonucleotides cloned into plasmids was used to further test the effect of such structures on sequencing. Our analyses indicated that a stem–loop structure capable of G–T pairing mediated the orientation bias by stabilizing this structure for specific alleles in heterozygous situations. We propose that this wobble‐like pairing hinders DNA polymerase passage on one strand while, on the complementary strand, the nonpaired A–C nucleotide counterparts allow unobstructed replication. Experimentation with synthetic amplicons that form similar stem–loop structures supported our hypothesis. We coined the term ‘replication diode’ for this effect and demonstrated that we can minimize it by lowering DNA and salt concentration. We also demonstrated that common genomic palindromes might induce the replication diode effect by applying bidirectional sequencing to an amplicon containing the palindrome within the human *miRNA 1‐1* gene. Hence, to obtain reliable peak‐height ratios, bidirectional sequencing should be practiced at the lowest possible ionic strength whenever estimating CNPs. Further research is needed to determine whether the observed variable stem–loop structures affect *PER2* regulation *in vivo*.

**Abbreviations**
HRM: high‐resolution melt
SNP: single‐nucleotide polymorphisms
Taq: *Thermus aquaticus*

Sanger sequencing is an important tool for the identification of genomic variations and it generally yields similar genotypes for allele variants irrespective of the direction of the sequencing reaction. Copy number proportions (CNPs) for allele variants in DNA sequences are important when studying genetic variability. Calculating peak‐height ratios of single‐nucleotide polymorphisms (SNPs) observed in sequencing chromatograms is a practical method for estimating their CNPs 1. This approach has been further implemented to infer short‐range linkage information 2 and haplotype alleles involved in copy number variation 3.

The Assaf and the Awassi sheep breeds differ in morphological and physiological traits such as prolificacy and seasonality. In chromosomal regions pinpointed by a genome‐wide association study that compared SNP‐allele frequencies, we studied genomic loci that distinguish these breeds by systematically comparing deep‐sequencing data of coding genes between Awassi and Assaf individuals 4. To verify nonsynonymous mutations and their Mendelian inheritance in the candidate genes, we Sanger‐sequenced additional sheep individuals, estimating CNPs of the allele variants. In the case of ovine period circadian‐clock 2 (*PER2*) gene, sequencing chromatograms displayed anomalous behavior in which DNA sequencing of an amplicon from different directions yielded different SNP results. Although such behavior has been observed by others (https://www.researchgate.net/post/Sequencing_DNA_from_different_directions_different_result_at_one_base, last access 12.04.17) the current literature poorly addresses the scientific reason underlying this observation. Aiming to resolve this important issue, we took advantage of the occurrence of three such adjacent SNPs to investigate this phenomenon. Based on our findings, we propose a mechanism in which the orientation dependency of the chromatogram peaks stems from an orientation‐dependent block that hinders the passage of the DNA polymerase.

## Materials and methods

### Native system

Genomic DNA was extracted and amplified as previously described 4. Briefly, template DNA was amplified using PCR primers (Table 1) and Bio‐X‐ACT™ Long kit (Bioline Ltd., London, UK) according to the manufacturer's instructions and the following conditions: 30 cycles for 40 s at 92 °C, 60 s at 63 °C, and 60 s at 68 °C. PCR products were separated on agarose gels, excised, and purified with AccuPrep^®^ Gel Purification Kit (BioNeer Corp., Seoul, Korea). Chromatograms were obtained by capillary ABI3730 sequencing (DNA Sequencing Unit, Biological Services, Weizmann Institute of Science, Rehovot, Israel) using a BigDye^®^ Terminator v1.1 Cycle Sequencing Kit (Applied Biosystems, Foster City, CA, USA). High‐resolution melt (HRM) analysis was performed in an Eco Real‐Time PCR System (Illumina, San Diego, CA, USA) using the KAPA™ HRM FAST PCR Kit (KAPA Biosciences, Woburn, MA, USA) following the assay conditions recommended by the manufacturer. mfold (Version 3.6 for Linux, 5) analyses were conducted using the 435‐bp amplicon sequences and settings that mimic the HRM analysis at 90 °C (NA = DNA, *T* = 90, NA_CONC = 0.88, MG_CONC = 0.003), or at the sequencing elongation temperature (60 °C) for the analysis of synthetic oligonucleotides and of the *miR1‐1* palindrome. Values of correlation and paired Student's *t* test were calculated using the Microsoft Excel^®^ software (Microsoft Corporation, Redmond, WA, USA).

**Table 1:** Nucleotide sequences used to analyze the ovine *PER2* gene and the replicational diode element

	Forward 5′→3′	Reverse 5′→3′
Short PCR amplicon: 435 bp	CACCGTCTCTCAAGGGACTC	ACCCTCCACCCCACTCAG
Long PCR amplicon: 531 bp	GTCCTGCCTCTGTGTCCACT	GGGTCTTCCAAACACACGTC
ATT haplotype sequence^a^	AGGGGCCGGT**A**GTCTGAGGAGCCTC**T**GTCCCCTCCCCCACCTCCACTTTGCAC**T**GAGACCTGAC	
ACG haplotype sequence	AGGGGCCGGT**A**GTCTGAGGAGCCTC**C**GTCCCCTCCCCCACCTCCACTTTGCAC**G**GAGACCTGAC	
GCG haplotype sequence	AGGGGCCGGT**G**GTCTGAGGAGCCTC**C**GTCCCCTCCCCCACCTCCACTTTGCAC**G**GAGACCTGAC	
LoopG^b^	aattTGGAGGAGGGGACGGC**G**GTCTGAGGAGCCAC**C**GCCCCCTCCCCCA	
LoopC	gatcTGGGGGAGGGGGC**G**GTGGCTCCTCAGAC**C**GCCGTCCCCTCCTCCA	
LoopA	aattTGGAGGAGGGGACGGC**A**GTCTGAGGAGCCAC**A**GCCCCCTCCCCCA	
LoopT	gatcTGGGGGAGGGGGC**T**GTGGCTCCTCAGAC**T**GCCGTCCCCTCCTCCA	
VectorF	GCAAGTGTAGCGGTCACG	
VectorR (M13 reverse)	CAGGAAACAGCTATGAC	
miR1F	TGTAAAACGACGGCCAGTCGGCGTCCCGGGGTCTTGGAACTGCATGCAGACTGCCTGCTTGG	
miR1variantF	TGTAAAACGACGGCCAGTCGGCGTCCCGGGGTCTTGGAACTGCATGCAGGGTTCCGGCTTGG	
miR1R	ACACGACCGTCCACCAAC	
M13F	TGTAAAACGACGGCCAGT	

a Nucleotides that form the haplotype variation are underlined and boldfaced.

b Lower case denotes the sticky ends that mediate the *Eco*RI–*Bam*HI ligation.

### Synthetic system

Oligonucleotides pairs (0.5 nmole·μL^−1^, each) were annealed at room temperature (LoopG with LoopC; LoopA with LoopT, Table 1). Plasmids (pBR322 ori) were constructed by inserting the annealed oligonucleotides into *Eco*RI–*Bam*HI sites; and transformed into homemade Top10 competent cells. Clones were mini‐prepped using a PureYield Plasmid Miniprep System kit (Promega, Madison, USA) and sequenced on an ABI3500 genetic analyzer (DNA Sequencing Unit, Tel‐Aviv University, Tel‐Aviv, Israel) using a BigDye^®^ Terminator v1.1 Cycle Sequencing Kit (Applied Biosystems) and primers (VectorF, VectorR, Table 1). Similarly, the mixed plasmid (1 : 1) DNA was amplified using these primers and the KAPA HiFi HotStart ReadyMix (Kapa Biosystems, Wilmington, MA, USA) according to the manufacturer's instructions and the following conditions: 30 cycles for 30 s at 98 °C, 20 s at 63 °C, and 10 s at 72 °C. PCR products were separated on agarose gels, excised, purified, and sequenced using the procedure described for the plasmid isolation. For testing effects of buffer molarity on sequencing results DNAs were suspended in Tris‐HCl buffer with increasing molarity (0, 40, 80 mm). The melting temperature (*T*_m_) of the stem–loop structure formed by each of the LoopG, LoopC; LoopA and LoopT oligonucleotides was determined by HRM analysis using reactions of total volume of 20 μL and 2–8 μm single‐strand oligonucleotide suspended in 1× Fast SYBR^®^ Green Master Mix (Applied Biosystems).

### Analysis of human miR1 palindrome

To facilitate sequencing and to introduce detectable variation, the design of the forward oligonucleotide primers (62 bp) was extended to include the M13 forward primer sequence (miR1F, miR1variantF, Table 1). Following the procedures described in the section Native system, human DNA was extracted, amplified, sequenced, and analyzed using the reverse miR1R primer (Table 1). To examine the replication diode effect, normal and variant PCR products (264 bp) were gel‐purified, mixed (1 : 1; 1.8), and sequenced using M13F and miR1R oligonucleotide primers (Table 1). Sequencing at high ionic strength was mediated by addition of NaCl (60 mm).

## Results

On chromosome 1, we encountered the period circadian‐clock 2 gene (*PER2*, GenBank accession number: XM_012180114), which includes five exons. The 3′‐end exon encodes the conserved domain of the period protein 2/3C‐terminal region (GenBank accession number: XP_012035504). At its putative 5′‐end, we found adjacent SNPs coding for nonconservative amino‐acid substitutions (S9G, C14R, L22R), which formed three haplotypes (Table 1). The DNA‐seq results showed that the Assaf individual was homozygous for the ATT haplotype, while the Awassi individual was heterozygous for the ACG/GCG haplotypes.

**Figure 1:**
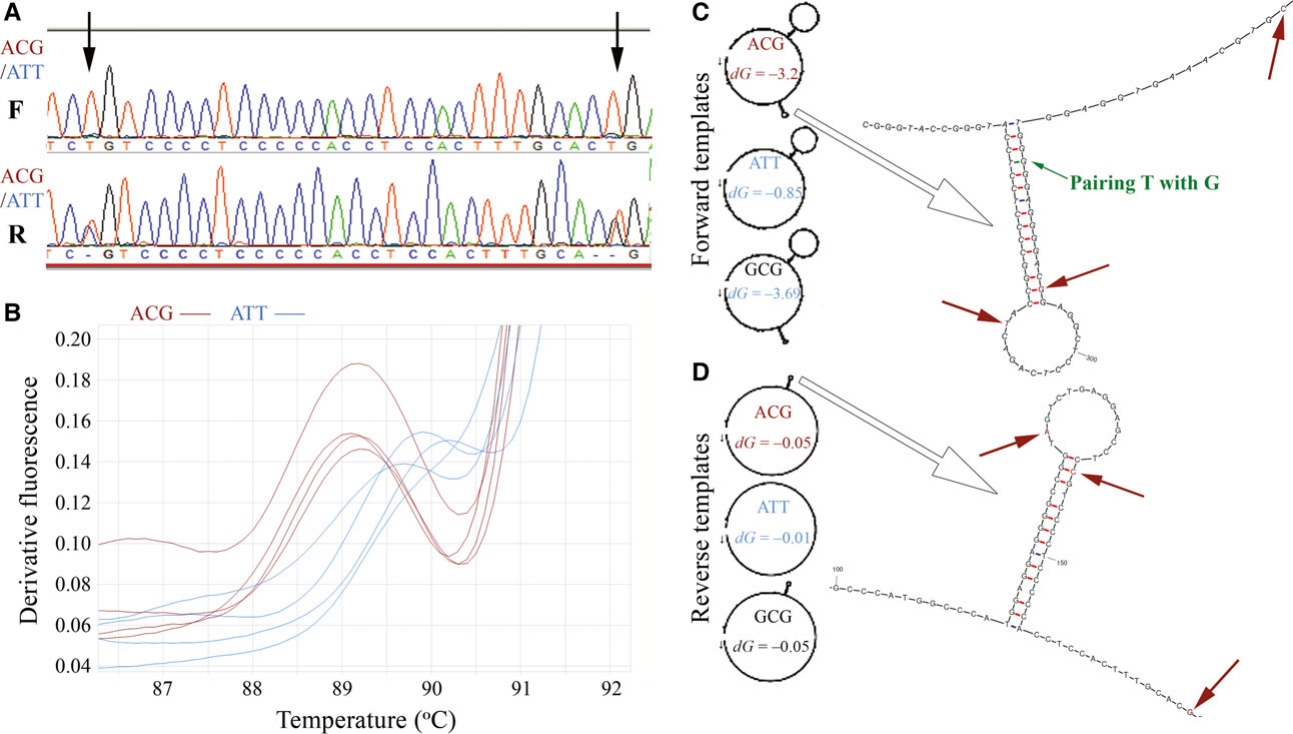
Sequencing heterozygous template DNA from different directions yields different results. (A) Chromatograms of the 531‐bp *PER2* amplicon sequenced from the forward (F) or reverse (R) direction by capillary using BigDye^®^ Terminators. The presented DNA template was of a sheep heterozygote carrying ACG and ATT haplotypes. Arrows point to the locations of the second and third haplotype SNPs.(B) HRM analysis of the 435‐bp *PER2* amplicon. Derivative curves of four ACG‐haplotype homozygotes (brown) were compared to those of four ATT‐haplotype homozygotes (blue). (C) MFOLD prediction of the secondary structure of DNA templates that anneal to the forward primer. MFOLD analyses were conducted with the 435‐bp amplicon sequences and settings that mimic the HRM analysis at 90 °C. The free‐energy values (dG) for each fold are indicated within the template delineations. Small arrows indicate the annealed primers and the direction of DNA synthesis. (D) Secondary structure of DNA templates that anneal to the reverse primer. From the positions of stem–loop structures on the template delineations, large arrows point toward enlarged and detailed images of these structures, which are part of the MFOLD output. The complete images of the folded amplicons are given in Figs S1–S6. Other arrows indicate the exact position of these *PER2* SNPs. Base‐pairing is color‐coded as follows: AT/TA blue, CG/GC red, and GT/TG green.

Since seasonality of reproduction differs between these breeds and the circadian clock may affect this trait 6, we further validated this polymorphism by Sanger sequencing of 10 additional individuals from each breed (Fig. 1A). Using conventional procedures, we sequenced a PCR amplicon in the forward direction (435 bp, Table 1). This confirmed the prevalence of the ATT, ACG, and GCG haplotypes (50%, 40%, and 10%, respectively) in both the Awassi and Assaf populations. Surprisingly, all chromatogram traces obtained from the 20 sequenced individuals were homozygous, which did not fit (*P* < 10^−5^) the Hardy–Weinberg equilibrium. Repeated sequencing with different primer‐annealing temperatures (55 °C, 60 °C) or using other PCR primers in all combinations (Table 1) yielded similar results.

As deep‐sequencing did indicate the existence of a heterozygous state, we looked for further evidence of heterozygosity of the *PER2* SNPs by a BLAST search of 100 Moroccan sheep genomes (~ 10‐fold coverage each, GenBank accession number: BioProject PRJEB3137) against the ACG haplotype sequence (Table 1). Association of significant hits (*E*‐value < *e* ^−15^) with the three haplotypes resulted in valid genotypes (> 4 hits) for 86 individuals. Two haplotypes, ACG and ATT, were common in the Moroccan sheep, with frequencies of 0.58 and 0.42, respectively, and there was a single occurrence of the GCG haplotype. Genotype frequencies of 32 ACG/ACG, 18 ATT/ATT, and 35 ACG/ATT confirmed the expected Hardy–Weinberg equilibrium. Hence, we concluded that the ovine *PER2* gene was Mendelian inherited with no obvious structural variation, and that the Sanger sequencing results from the Assaf and Awassi populations that suggested otherwise were skewed.

Indeed, repeating the Sanger sequencing in the reverse direction yielded heterozygous genotypes for half of the ‘homozygous’ ATT carriers (Fig. 1A). As we hypothesized that secondary structures may hinder the passage of the *Thermus aquaticus* (Taq) DNA polymerase and cause this inconsistency between forward and reverse sequencing, we analyzed the short amplicon containing the ATT and ACG haplotypes (Table 1) using HRM analysis (Fig. 1B), which can reveal secondary DNA structures. The HRM‐derivative curve for the ACG haplotype peaked at 89.17 ± 0.05 °C, indicating its ability to form a stable fold at temperatures significantly lower (*P* < 0.03) than those needed by the ATT sequences, which peaked on average at 89.93 ± 0.15 °C. mfold software analysis 5 was used to assess these potential secondary structures (Fig. 1C–D). Free‐energy values for DNA templates that annealed to the forward primer indicated that G–T pairing promotes the formation of more stable stem–loop structures involving the *PER2* haplotypes’ SNPs than the complementary strand, which annealed to the reverse primer. This suggested that sequencing heterozygotes from the forward primer would preferentially target the ATT haplotype, in which the stem–loop structure does not slow the DNA replication (Fig. 1C). Thus, wobble‐like pairing between G and T nucleotides may have produced a ‘replication diode’ by hindering the passage of the DNA polymerase through stabilization of stem–loop structures in one direction, while in the other direction, the complementary strand remained available to replicate as the A–C nucleotide counterparts did not produce these structures.

### Testing a synthetic system using a different sequence

**Figure 2:**
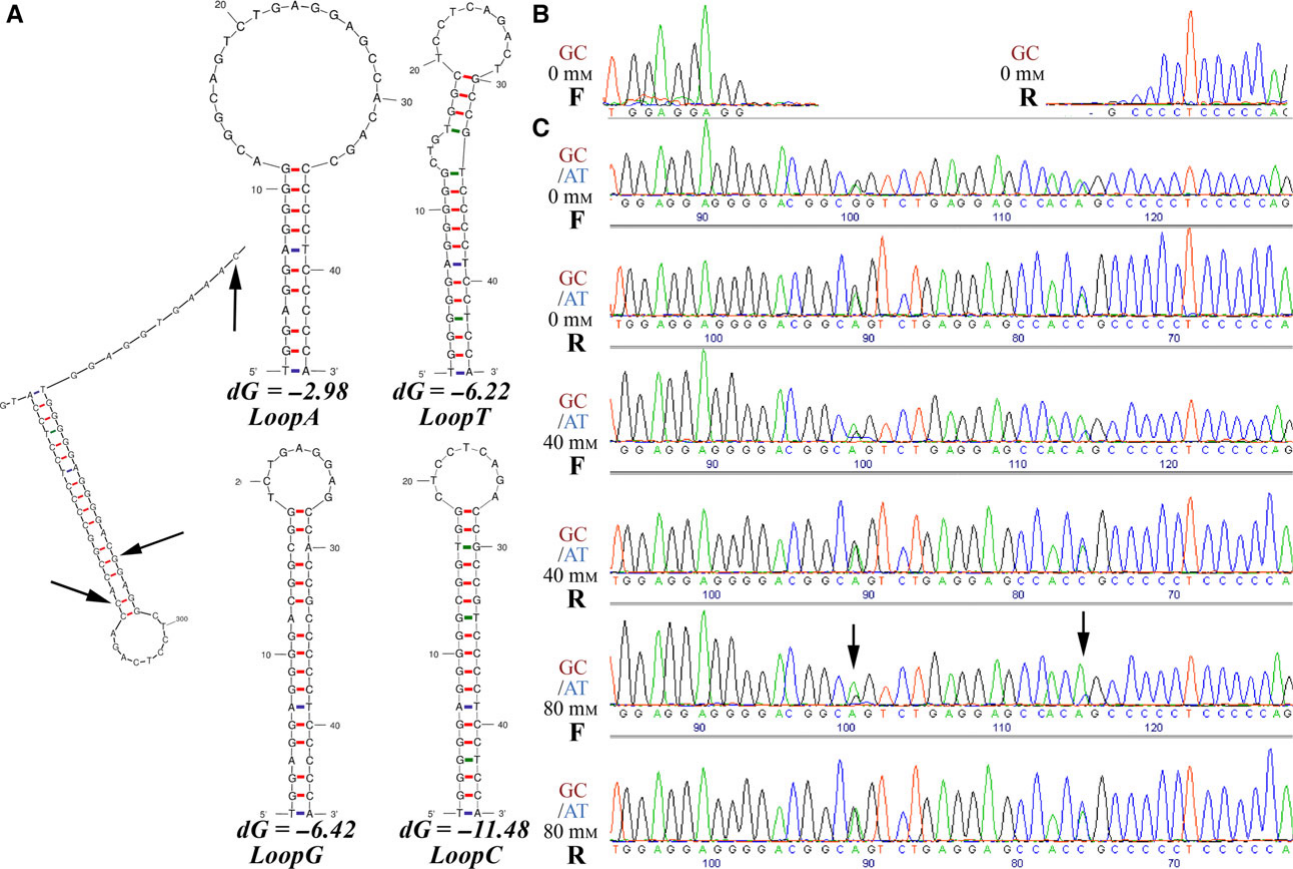
A synthetic system for analyzing the GCG‐haplotype like replication diode. (A) On the left, this panel presents an enlarged and detailed image of the lower structure observed in the GCG forward template, which is part of the MFOLD output in Fig. 1C. Arrows indicate the exact position of the *PER2* SNPs. To the right, this panel presents the MFOLD folds (*T* = 60 °C) of two pairs of complimentary oligonucleotides that form structures similar to that of the ATT and the GCG haplotypes, with enhanced G–T pairing. (B) Plasmid sequencing. The annealed oligonucleotides LoopA and T (AT); and LoopG and C (GC) were inserted into a sequencing vector and transformed clones were mini‐prepped and plasmid DNA (25 ng·μL^−1^) was bidirectionally sequenced with the vector sequencing primers (F, R). (C) Sequencing of PCR products. The plasmid mix (1 : 1) was PCR amplified using the vector sequencing primers and the purified amplification products were bidirectionally sequenced with these primers. The molarity of the Tris‐HCl buffer used for the suspension of the purified products is indicated (0–80 mm). Arrows point to the double peaks for which the effect of the replication diode was most pronounced.

To corroborate the replication diode hypothesis, we tested if a synthetic system would show the same effect. We designed two pairs of complimentary oligonucleotides with predicted 45‐bp stem–loop structures that were similar to that of the ATT and the GCG haplotypes (Table 1, Fig. 2A). These oligonucleotide pairs (AT and GC) included two positions analogous to the polymorphic sites of these haplotypes although their nucleotide sequence was not identical to the above described stem–loop structures. In the stem structure, we introduced two additional G–T pairing sites enhancing the possible effect of this pairing type (green bars, Fig. 2A). The annealed pairs were ligated into a cloning vector and clones were sequenced from both directions. Sequencing of the AT plasmid proceeded with no hindrance, while, the sequence of the GC plasmid was disrupted by a possible secondary structure next to the primer (Fig. 2B). Using these plasmids (1 : 1 mix) as a PCR template, we amplified and sequenced a 243‐bp amplicon, which when suspended in buffer with increasing molarity gradually reproduced the replication diode effect as pointed to by the base ratio in the double peaks (Fig. 2C).

#### HRM analysis of the synthetic system

**Figure 3:**
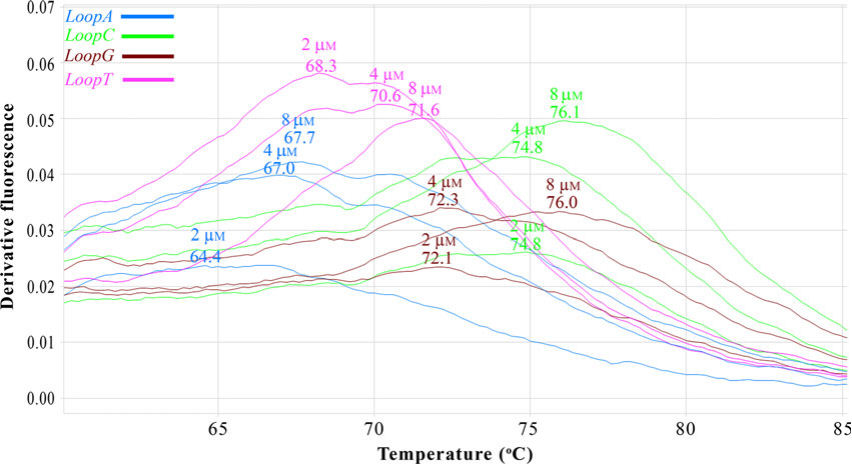
Determination of melting temperatures of stem–loop structures involved in the synthetic system for the replication diode. Using the melting curve obtained by HRM analysis, we determined the Tms of the single‐stranded oligonucleotides (LoopA, blue; LoopC, green; LoopG, brown; LoopT, pink). For each curve, the DNA concentration and the observed *T*_m_ is indicated at its highest peak.

Using the native amplicon (Fig. 1) the stem–loop structures had a different HRM peak temperature of 89.9 °C versus 89.1 °C, which made us wonder if such a small difference of < 1% would have a real‐world practical significance. Using HRM analysis, we better examined the local effect of the structures using the short 45‐bp oligonucleotides (Fig. 3). The observed Tms were dependent on the DNA concentrations and up to a ~4 °C difference was recorded for the same template in the examined range (2–8 μm). Elevated DNA concentrations correlated (*R* = 0.994) with increased Tms. Significant negative correlation (*R* = −0.901) was observed between the average Tms of each oligonucleotide and their predicted free energy (dGs) was effectively estimated by the current version of the mfold software, which can also account for wobble pairing (Fig. 2A). The Tms of the oligonucleotides with the wobble‐like pairing were significantly increased (*P* < 0.005) comparing to their nonwobble‐paired counterparts and up to a ~ 4 °C difference was recorded between these counterparts. Hence, both the ionic strength mediated by the DNA concentrations and wobble‐like pairing had significant effect on the observed Tms, which were in a range of ~ 64–76 °C. Taking into account that the elongation temperature in cycle sequencing is much lower (60 °C), these factors may indeed practically influence the sequencing processivity.

### Replication diode effect of the human miR1‐1 palindrome

**Figure 4:**
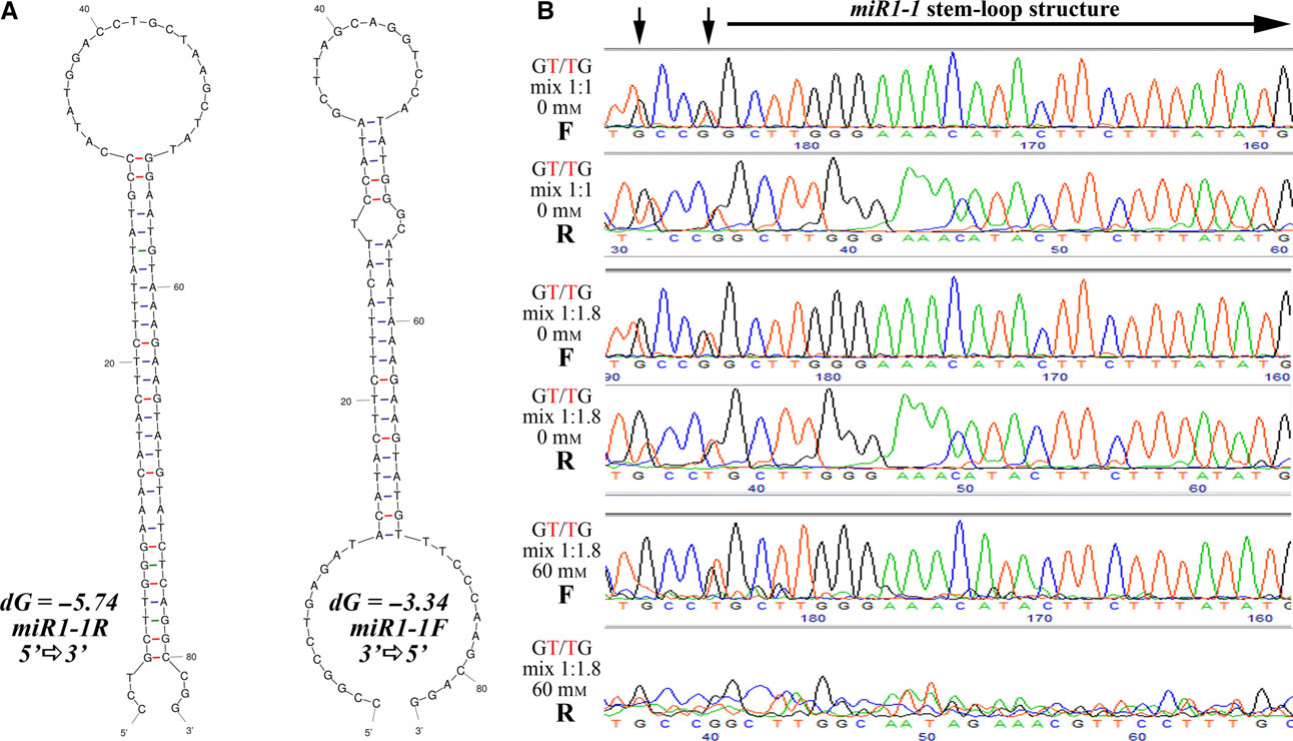
Replication diode effect in human *miR1‐1*. (A) MFOLD analysis (*T* = 60 °C) of the native sequence of the human *miR1‐1* palindrome. (B) Sequencing of PCR products. The amplicon mixes (1 : 1; 1 : 1.8) of normal and mutated amplicons were PCR amplified using the primers and the purified amplification products were bidirectionally sequenced using M13F and miR1R oligonucleotide primers (Table 1) under different conditions. Ionic strength mediated by addition of NaCl (60 mm). Vertical arrows point to the double peaks for which the effect of the replication diode was most pronounced. A horizontal arrow denotes the sequence of the left template shown in the previous panel. Supporting information including the nucleotide sequences and the MFOLD analysis for these PCR products is presented in Figs S7–S10.

To further demonstrate that common genomic palindromes may induce a replication diode effect, we applied bidirectional sequencing to an amplicon containing the palindrome within the human microRNA 1‐1 gene (*MIR1‐1*, GenBank accession number: NR_029780). In the 5′–3′ direction, mfold analysis indicated that this palindrome consists of 23 normal base‐pairings and that this palindrome was further stabilized by three wobble pairings (Fig. 4A). Folding of the complementary strand yielded no wobble pairing with an increase of ~ 72% in the free‐energy value (Fig. 4A). Introducing sequence variation at the lower part of the stem to enable visualization of the effect and further stabilization of this palindromic structure, we compare the sequence chromatograms of mixtures of normal and mutated amplicon at different ionic strengths (Fig. 4B). When mixed in a 1 : 1 ratio, the forward sequence template presented higher peak heights for mutated variant (variation T and G, Fig. 4B), whereas in the reverse orientation the higher peak heights were of the normal genotypes (variation G and T, Fig. 4B). When mixed in a 1 : 1.8 ratio, the forward orientation presented balanced peak heights for both variants, whereas the peak ratio was skewed toward the normal genotypes in the reverse orientation. Increasing ionic strength intensified this effect, indicating stabilization of the stem–loop structure that blocked the polymerase advancement only in the reverse orientation of both the normal and mutated amplicons.

## Discussion

The occurrence of three SNPs within 44 bp of the first exon of the ovine *PER2* gene provided a unique opportunity to investigate the unexplained phenomenon of Sanger sequencing that yields different results from different directions. This triplicate ensured that the observed orientation dependency of the chromatogram peaks is not sporadic but arises from a secondary structure that affects the whole amplicon, and produces an orientation‐dependent block that hinders the DNA polymerase's passage.

Conventional automated DNA sequencing, which was also used in this study, uses mutant Taq polymerase and dideoxynucleotide triphosphates (ddNTPs). In this sequencing, template bias has been mainly attributed to differential discrimination of this polymerase against ddNTPs, especially low binding of ddTTP 7, 8. The blocking of Taq polymerase by DNA secondary structures has been well documented on G‐rich DNA templates that are known to assume secondary structures stabilized by Hoogsteen base‐pairing between the G residues 9. However, the effect of DNA hairpin structures or wobble‐like pairing on Taq polymerase has been little studied.

Formation of hairpin structures during *in vivo* replication in humans has been extensively studied in cases of instability of CTG/CAG trinucleotide‐repeat sequences, which are responsible for human neurological or neuromuscular disorders 10. *In vitro* studies of replication of these repeats have shown that the orientation‐dependent blocking of the polymerase's passage affects progression on CAG and CTG repeats. This blockage was suggested to be due to the presence of wobble pairing on single‐stranded templates; the CTG forms more stable hairpin structures than CAG, but the passage of DNA polymerase is most strongly blocked when CAG is the template 11. This supports an alternative mechanism in which the orientation dependence of replication efficiency is influenced by differential processivity of the polymerase on the primary templates. Nevertheless, preventing the formation of stem–loop structures by *in vitro* addition of single‐stranded, DNA‐binding protein eliminates the orientation‐dependent impediment to DNA polymerase's passage across the CAG/CTG repeats 11, hence, confirming the important role of stable hairpin in inducing the dependence of replication efficiency on orientation. The actual prediction of these structures’ stability should include additional factors, such as bulge loop size 12.

The relative low elongation temperature (60 °C) in cycle sequencing stabilizes DNA secondary structure and thus may explain why the similar techniques of PCR and deep‐sequencing were not biased. It should be noted that palindromic structure may be also stalled by intermolecular base‐paring and the importance of such interactions would increase in higher DNA concentrations. This alternative hypothesis suggesting that more complex interactions than that of the stem–loop structure may impede the DNA polymerase was supported by the observation that the diode effect and the Tms of the sequence motifs involved did increase in higher DNA concentrations. Yet, the simpler explanation remains that the increased ionic strength provided by the higher concentration of free counterions at the higher DNA concentration was in direct analogy to the increased protection to thermal denaturation afforded by increasing concentrations of added salt 13.

## Conclusions

The pronounced directional effect that we encountered in Sanger sequencing indicates that bidirectional sequencing should be practiced whenever estimating CNPs. However, in population studies this may double the cost. Therefore, when relying on unidirectional sequencing, avoiding amplicons capable of forming secondary structures would prevent the diode effect or when this is not possible the lowering of DNA and salt concentration may reduce this effect. Thus, reducing the ionic strength of the sequencing reaction may be a tool to destabilize secondary structures that impede the DNA polymerase. Further research is needed to examine whether the observed variable stem–loop structures affect *in vivo* regulation of *PER2*. In *in vivo*, such structures may form cruciform, which are a common DNA feature important for regulating biological processes 14.

## Author contributions

AS performed and interpreted the sequencing and HRM analyses; US performed the experiments on the synthetic system; EG contributed sheep samples and conceived and designed experiments; ES conceived and designed experiments and performed the bioinformatic analyses; All authors wrote, read, and approved the article.

## Supporting information


**Fig. S1.** MFOLD output, the complete image of the folded forward template of the amplicon of ACG haplotype.
**Fig. S2.** MFOLD output, the complete image of the folded forward template of the amplicon of ATT haplotype.
**Fig. S3.** MFOLD output, the complete image of the folded forward template of the amplicon of GCG haplotype.
**Fig. S4.** MFOLD output, the complete image of the folded reverse template of the amplicon of ACG haplotype.
**Fig. S5.** MFOLD output, the complete image of the folded reverse template of the amplicon of ATT haplotype.
**Fig. S6.** MFOLD output, the complete image of the folded forward template of the amplicon of GCG haplotype.
**Fig. S7.** MFOLD output, the complete image of the folded forward template (5′–3′) of the amplicon of miR1‐1 fused to M13 primer.
**Fig. S8.** MFOLD output, the complete image of the folded reverse template (3′–5′) of the amplicon of miR1‐1 fused to M13 primer.
**Fig. S9.** MFOLD output, the complete image of the folded forward template (5′–3′) of the mutated amplicon of miR1‐1 fused to M13 primer.
**Fig. S10.** MFOLD output, the complete image of the folded reverse template (3′–5′) of the mutated amplicon of miR1‐1 fused to M13 primer.
