# Supplementary material for: Sequence motifs capable of forming DNA stem–loop structures act as a replication diode
Source: FEBS Open Bio. 2017 Jun 4;7(7):944–52. doi: 10.1002/2211-5463.12233 (PMC5494291; doi:10.1002/2211-5463.12233)
Supplement: Supplementary file 1 — Fig. S1. MFOLD output, the complete image of the folded forward template of the amplicon of ACG haplotype. Fig. S2. MFOLD output, the complete image of the folded forward template of the amplicon of ATT haplotype. Fig. S3. MFOLD output, the complete image of the folded forward template of the amplicon of GCG haplotype. Fig. S4. MFOLD output, the complete image of the folded reverse template of the amplicon of ACG haplotype. Fig. S5. MFOLD output, the complete image of the folded reverse template of the amplicon of ATT haplotype. Fig. S6. MFOLD output, the complete image of the folded forward template of the amplicon of GCG haplotype. Fig. S7. MFOLD output, the complete image of the folded forward template (5′–3′) of the amplicon of miR1‐1 fused to M13 primer. Fig. S8. MFOLD output, the complete image of the folded reverse template (3′–5′) of the amplicon of miR1‐1 fused to M13 primer. Fig. S9. MFOLD output, the complete image of the folded forward template (5′–3′) of the mutated amplicon of miR1‐1 fused to M13 primer. Fig. S10. MFOLD output, the complete image of the folded reverse template (3′–5′) of the mutated amplicon of miR1‐1 fused to M13 primer. [file FEB4-7-944-s001.pdf]

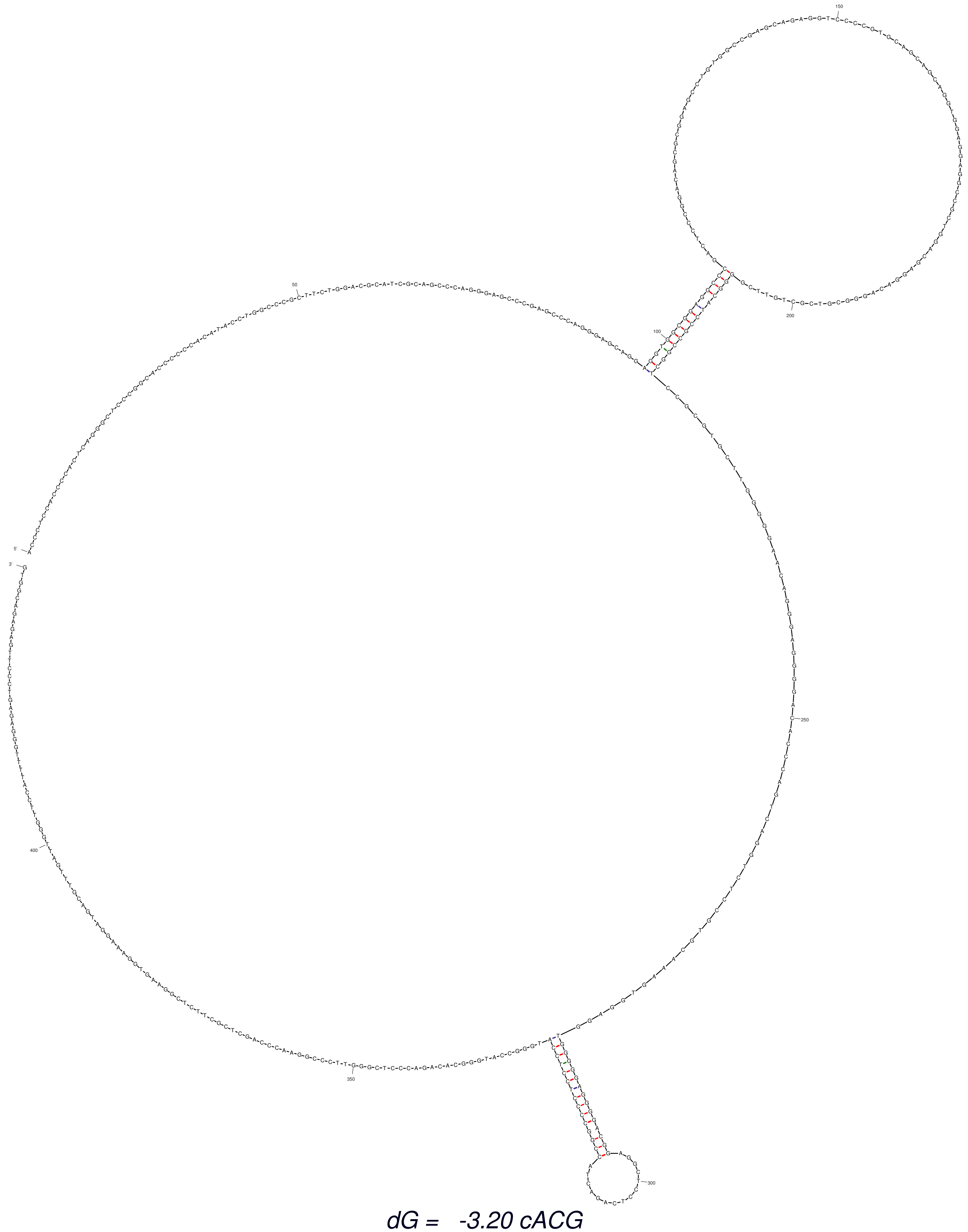

dG = -3.20 cACG

Fig. S1: MFOLD output,the complete image of the folded forward template of the amplicon of ACG haplotype

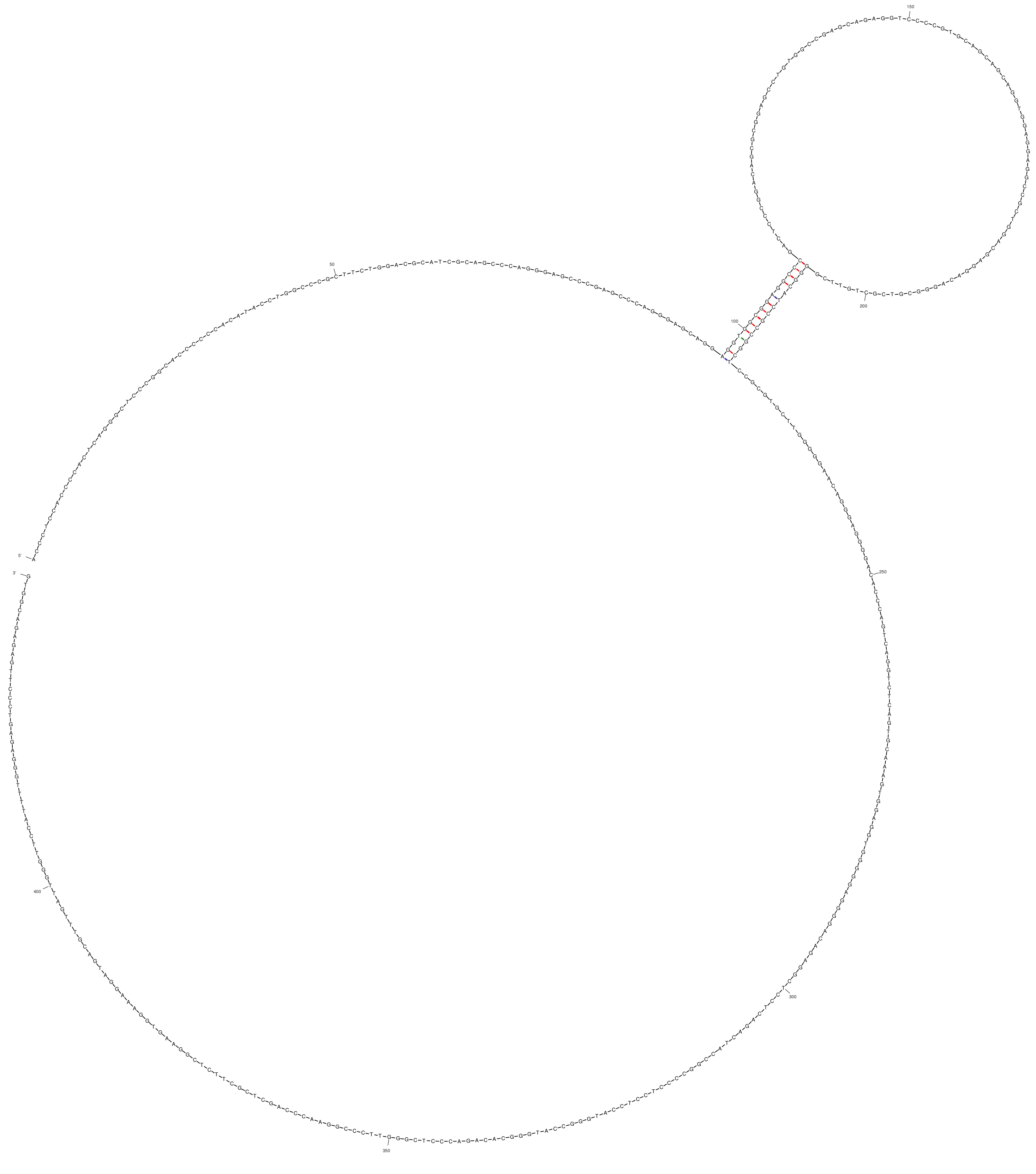

Fig. S2: MFOLD output,the complete image of the folded forward template of the amplicon of ATT haplotype

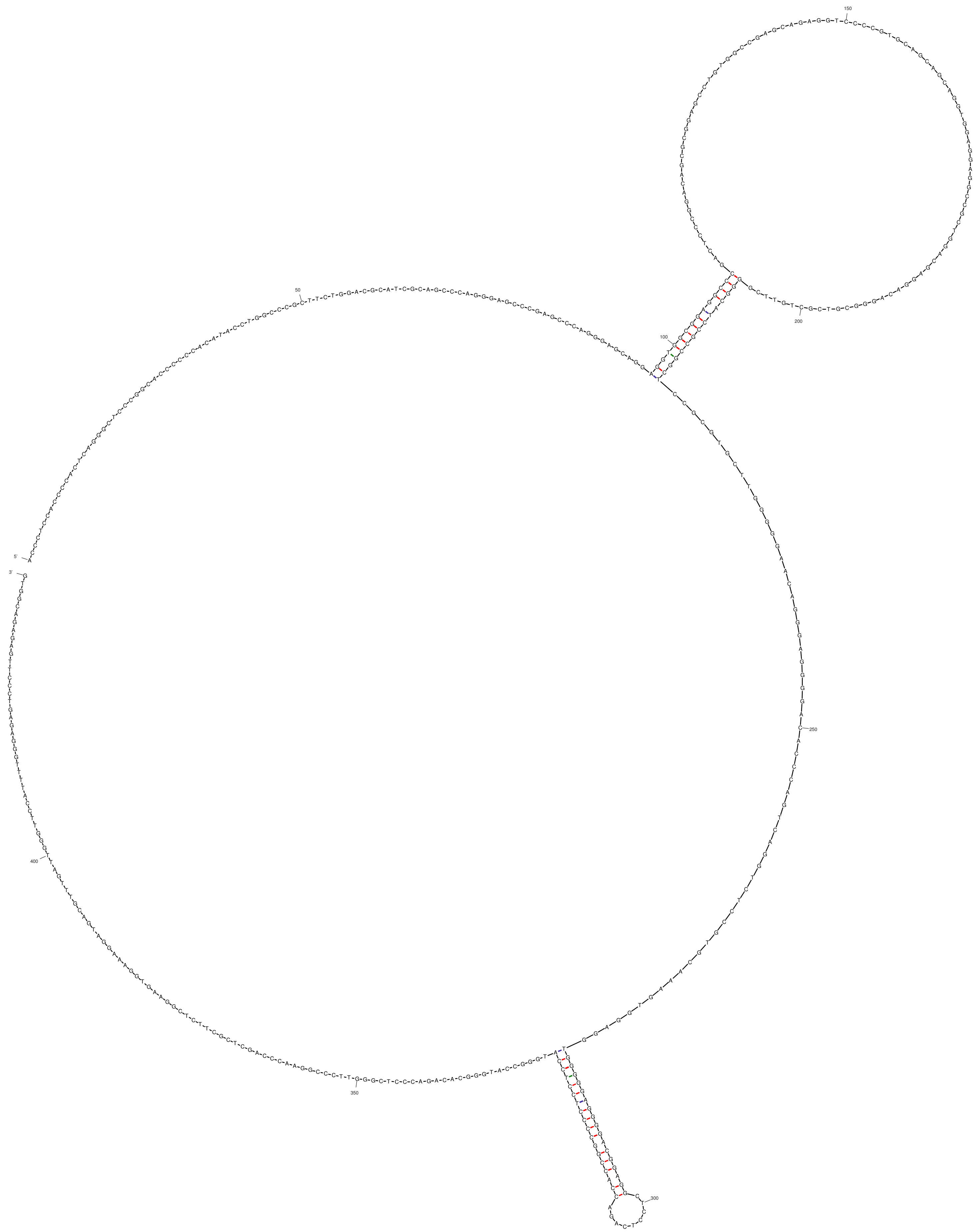

dG = -3.69 cGCG

Fig.S3: MFOLD output,the complete image of the folded forward template of the amplicon of GCG haplotype

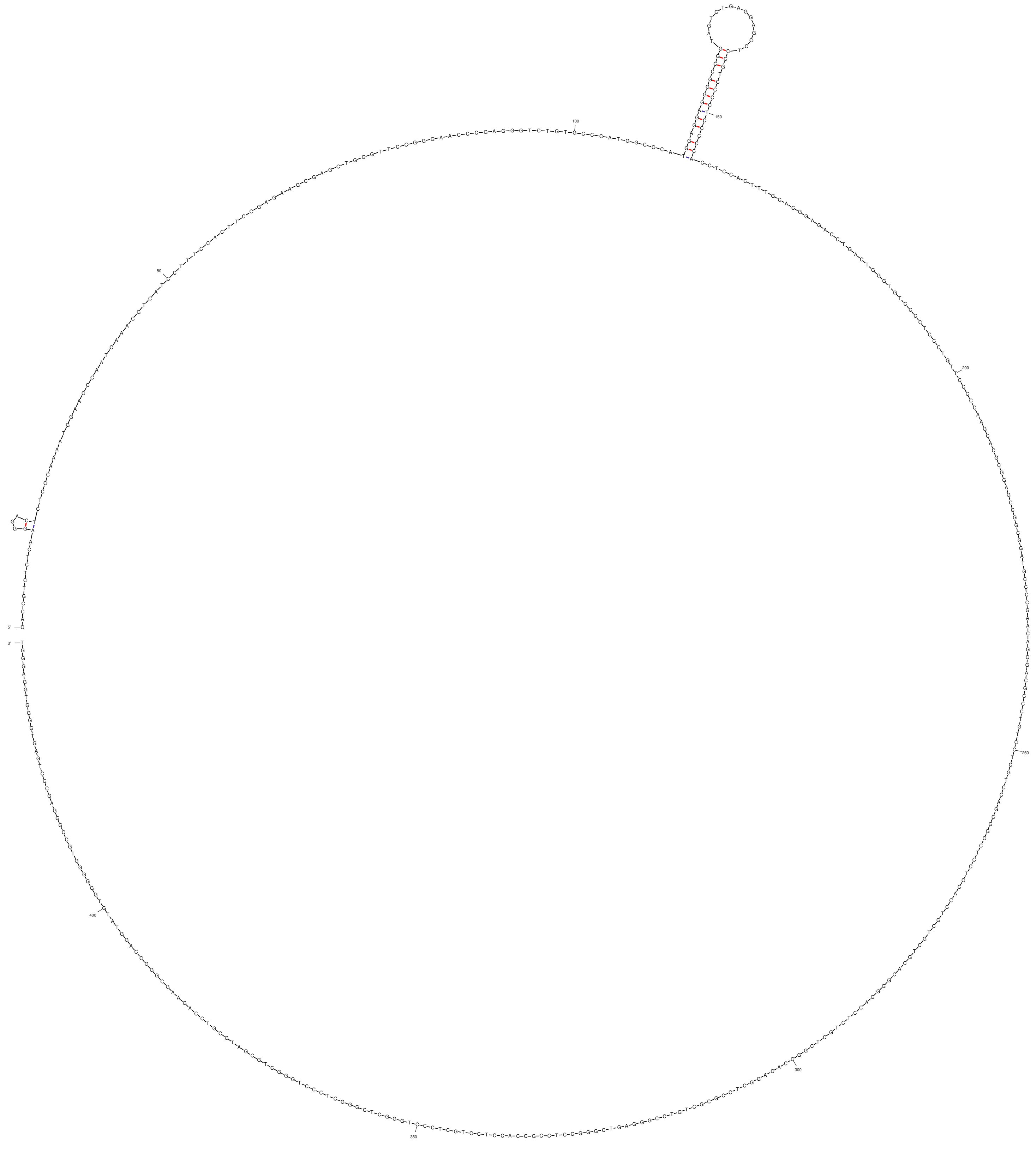

dG = -0.05 ACG

Fig. S4: MFOLD output,the complete image of the folded reverse template of the amplicon of ACG haplotype

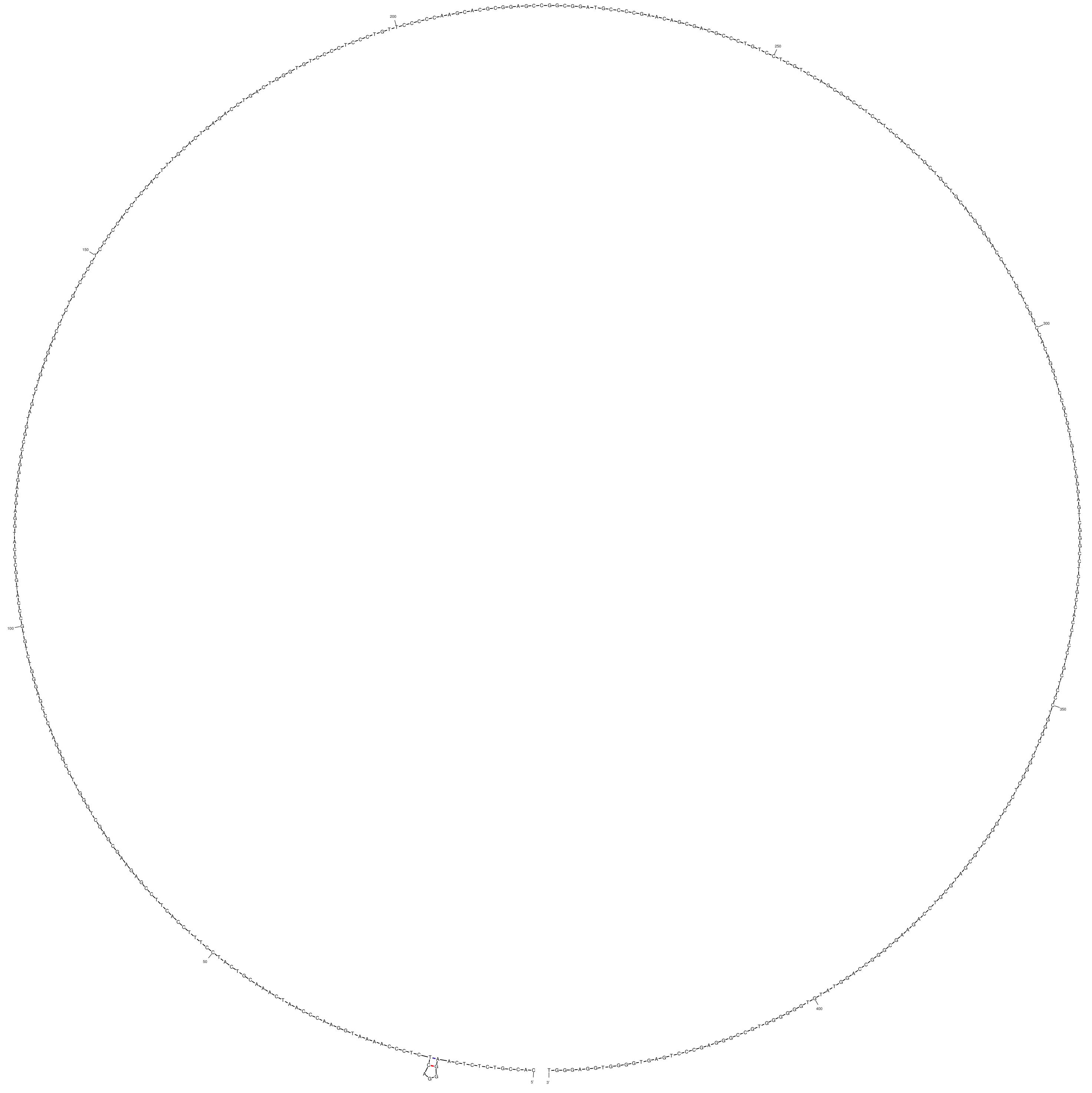

dG = -0.01 ATT

Fig. S5: MFOLD output,the complete image of the folded reverse template of the amplicon of ATT haplotype

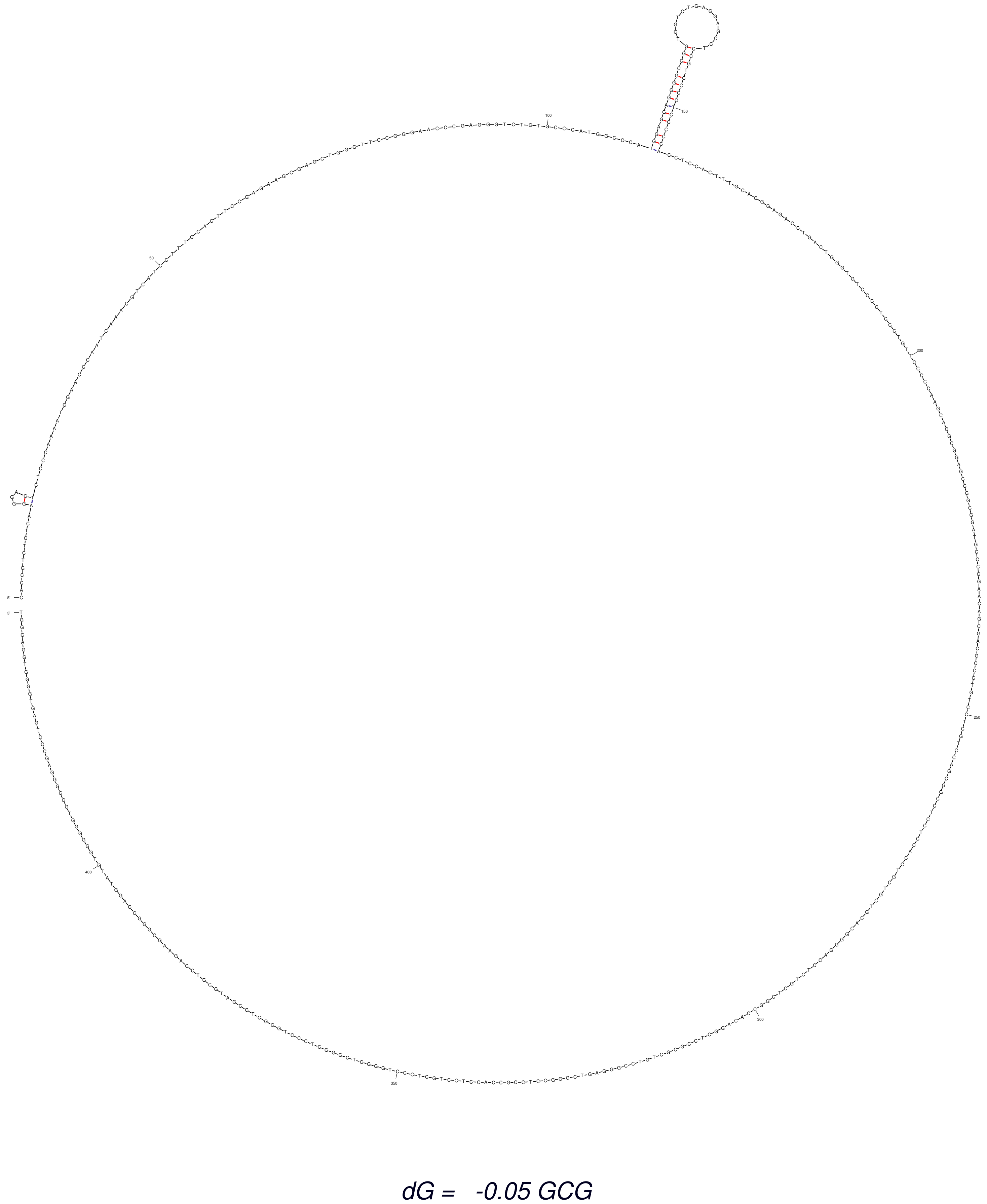

Fig.S6: MFOLD output,the complete image of the folded reverse template of the amplicon of GCG haplotype

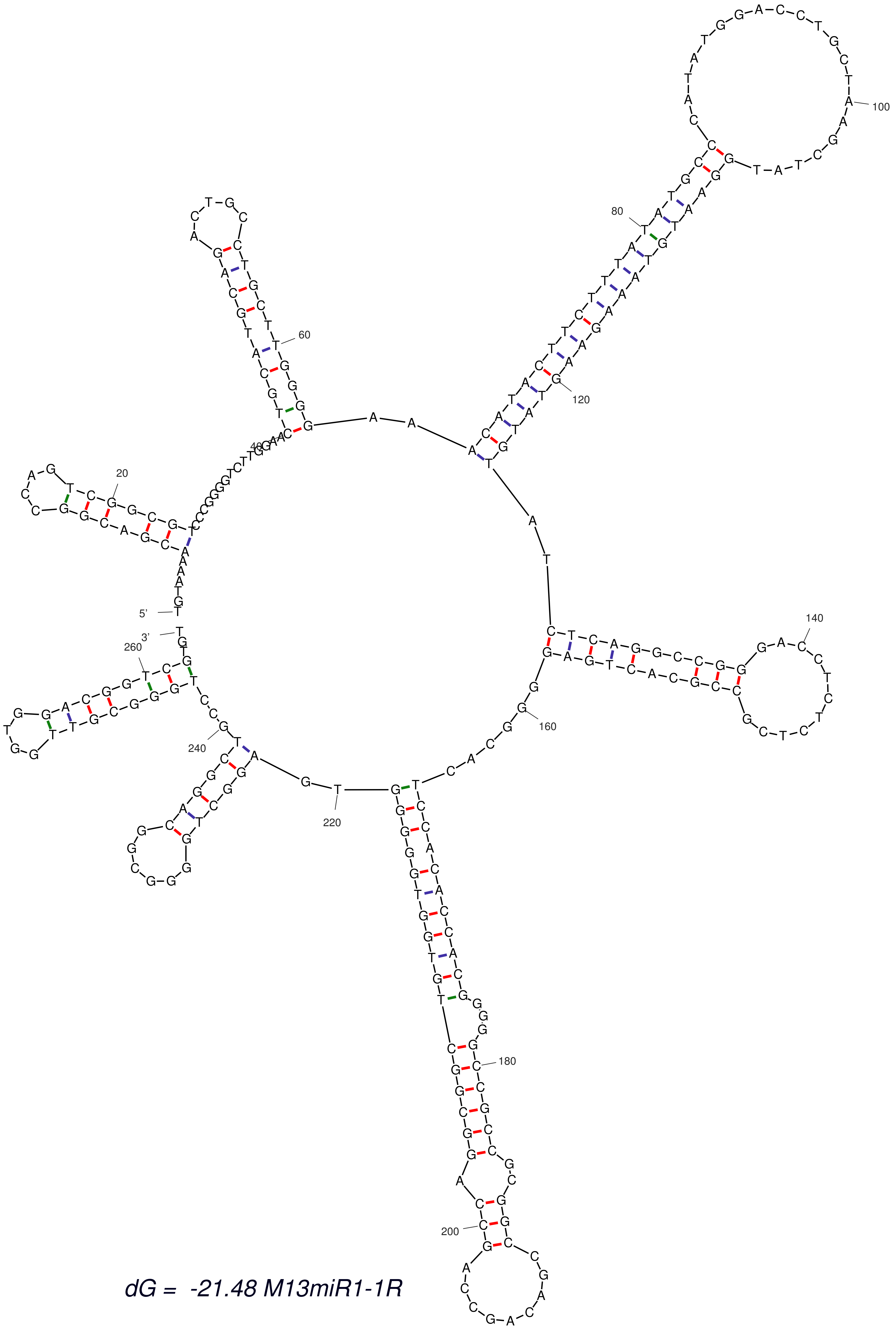

Fig. S7: MFOLD output, the complete image of the folded forward template (5' to 3') of the amplicon of miR1-1 fused to M13 primer.

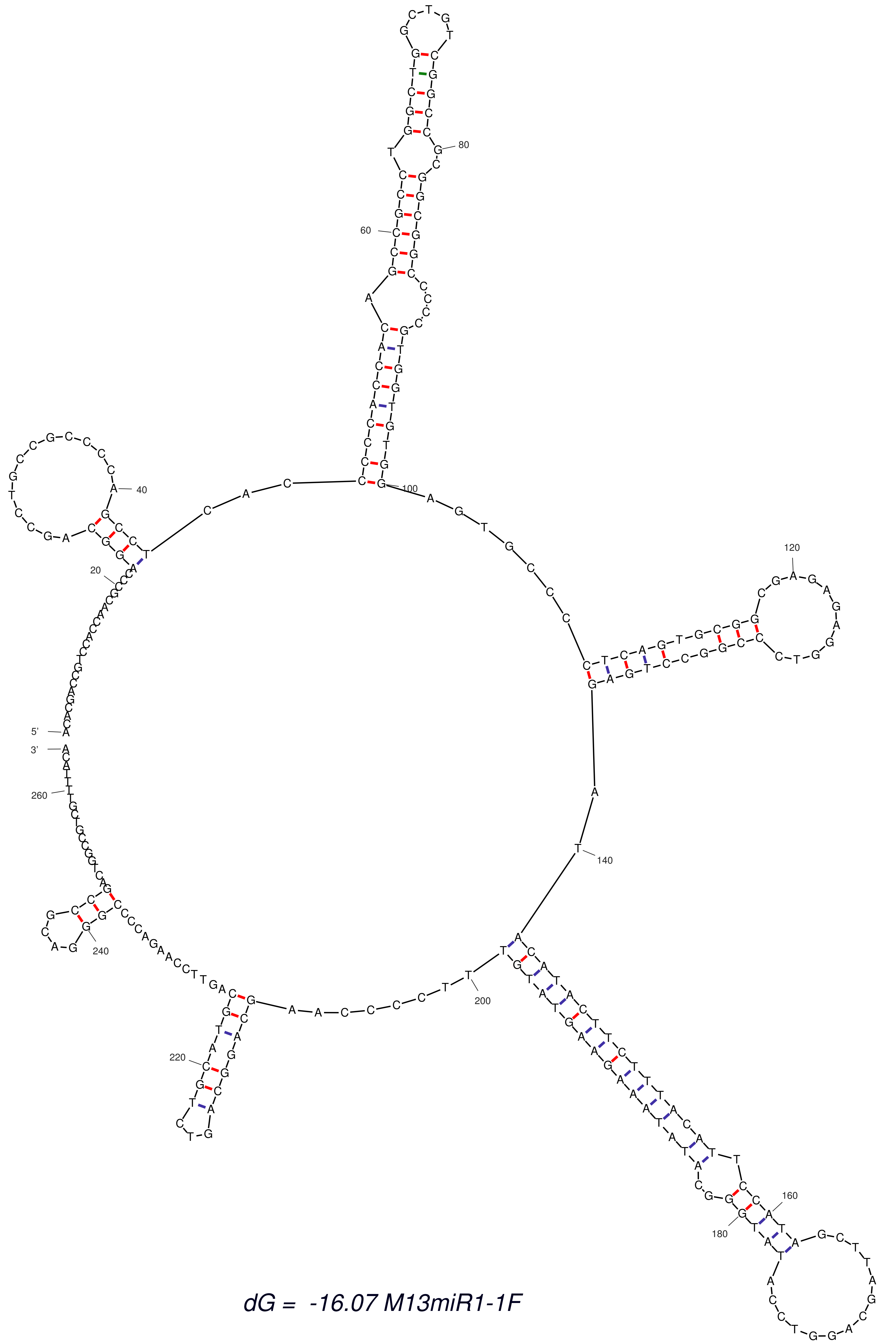

Fig. S8: MFOLD output, the complete image of the folded reverse template (3' to 5') of the amplicon of miR1-1 fused to M13 primer.

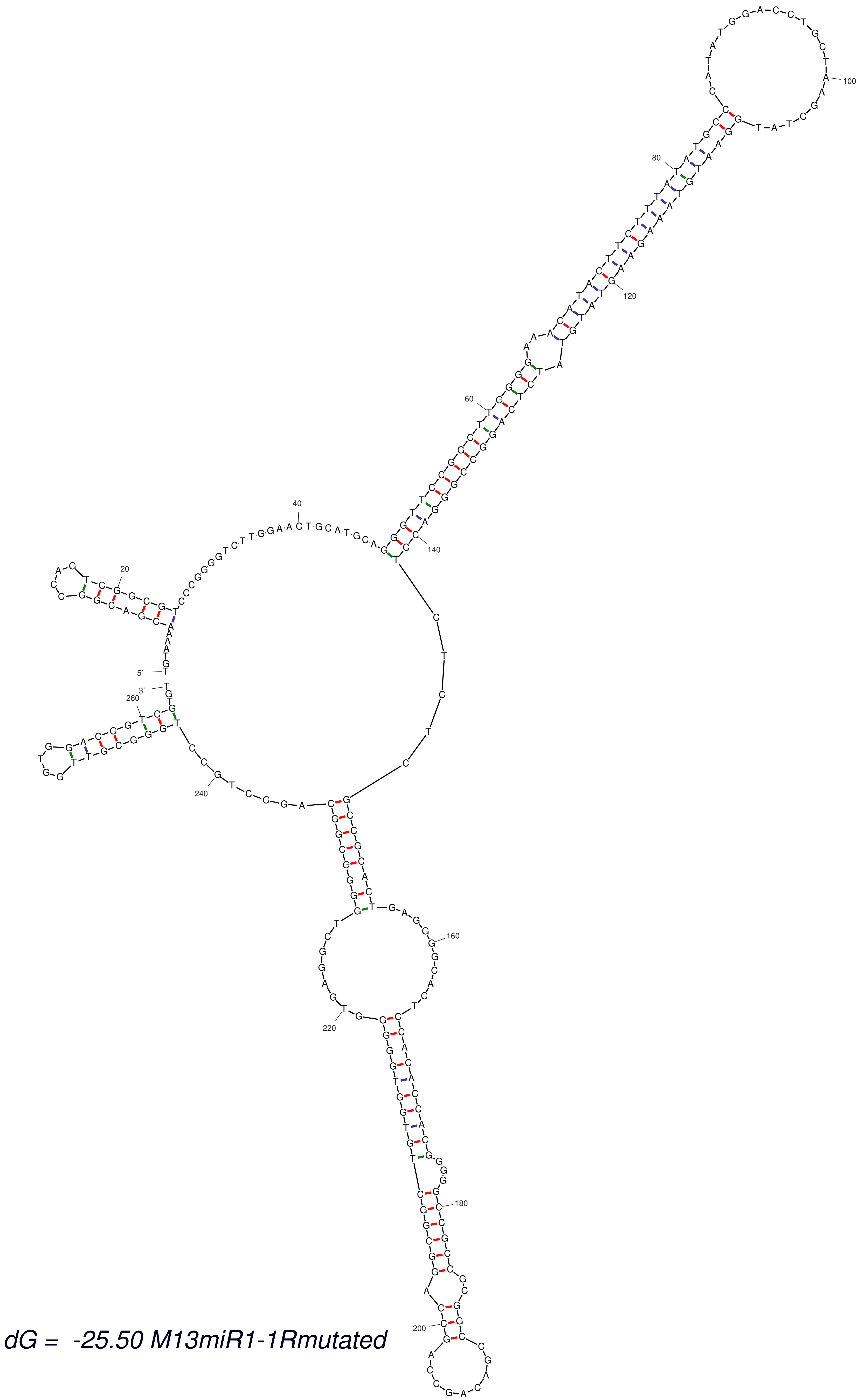

Fig. S9: MFOLD output, the complete image of the folded forward template (5' to 3') of the mutated amplicon of miR1-1 fused to M13 primer.

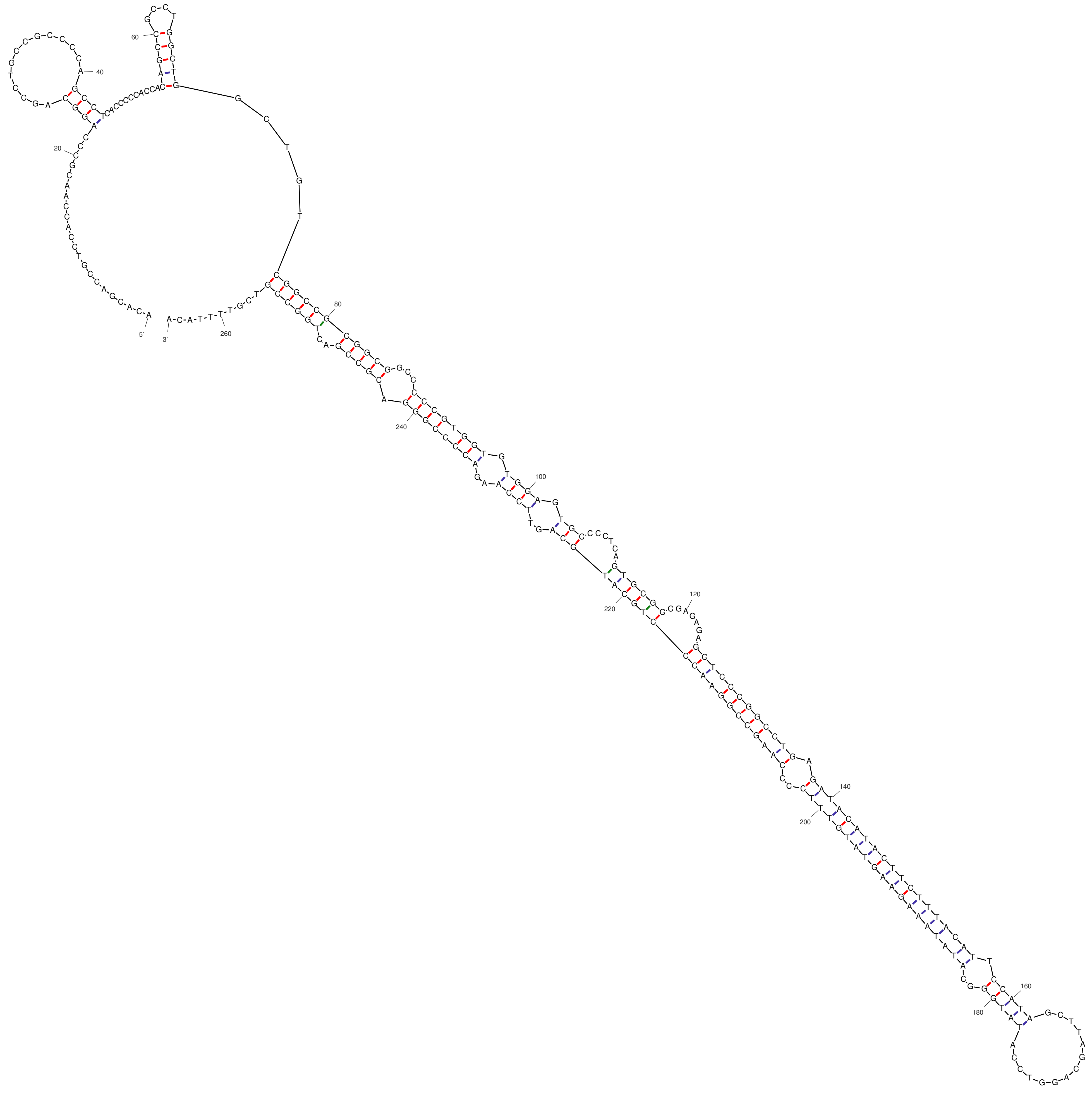

dG = -16.75 M13miR1-1Fmutated

Fig. S10: MFOLD output, the complete image of the folded reverse template (3' to 5') of the mutated amplicon of miR1-1 fused to M13 primer.
